# Supplementary material for: Heat adaptation of phage T7 under an extended genetic code
Source: Virus Evol. 2021 Dec 1;7(2):veab100. doi: 10.1093/ve/veab100 (PMC8923235; doi:10.1093/ve/veab100)
Supplement: veab100_Supp [file veab100_supp.zip › Sequencing_Analysis.zip!/Sequencing_Analysis/A2/evidence/JC_50.html]

BRESEQ :: Evidence


| New junction evidence | | | | | | | | | | | |
| --- | --- | --- | --- | --- | --- | --- | --- | --- | --- | --- | --- |
|  | | seq id | position | reads (cov) | reads (cov) | score | skew | freq | annotation | gene | product |
| \* | ? | NC\_001604.1 | = 13277 | 205 (0.450) | 231 (0.520) | 119/586 | 1.3 | 69.5% | intergenic (–/–) | *–/–* | –/– |
|  | ? | NC\_001604.1 | 14297 = | 3 (0.010) |  |  |  |  | intergenic (–/–) | *–/–* | –/– |

Only 100 of 243 total aligned reads displayed.  

`GACAAAGGTAAAGCACATGAGGAAGGTCGCCCCGTTTCTATTACTGACCTACGTGGTTCTGGCGCACTACGCCAACTATCTGATACTATTATTGCCCTTGAGCGTAATCAGCAAGGCGATATGCCTAACCTTGTCCTCGTTCGTATTCTCAAGTGCCGCTTTACTGGTGATACTGGTATCGCTGGCTACATGGAATACAACAAGGAAACCGGATGGCTTGAACCATCAAGTTACTCAGGGGAAGAAGAGTCACACTCAGAGTCAACAGACTGGTCCAACGACACTGACTTCTGACAGGATTCTTGA‑‑‑‑‑‑‑‑‑‑‑‑‑‑‑‑‑‑‑‑‑‑‑‑‑‑‑‑‑‑‑‑‑‑‑‑‑‑‑‑‑‑‑‑‑‑‑‑‑‑‑‑‑‑‑‑‑‑‑‑‑‑‑‑‑‑‑‑‑‑‑‑‑‑‑‑‑‑‑‑‑‑‑‑‑‑‑‑‑‑‑‑‑‑‑‑‑‑‑‑‑‑‑‑‑‑‑‑‑‑‑‑‑‑‑‑‑‑‑‑‑‑‑‑‑‑‑‑‑‑‑‑‑‑‑‑‑‑‑‑‑‑‑‑‑‑‑‑‑‑‑‑‑‑‑‑‑‑‑‑‑‑‑‑‑‑‑‑‑‑‑‑‑‑‑‑‑‑‑‑‑‑‑‑‑‑‑‑‑‑‑‑‑‑‑‑‑‑‑‑‑‑‑‑‑‑‑‑‑‑‑‑‑‑‑‑‑‑‑‑‑‑‑‑‑‑‑‑‑‑‑‑‑‑‑‑‑‑‑‑‑‑‑‑‑‑‑‑‑‑‑‑‑‑‑‑‑‑‑‑‑‑‑‑‑‑‑‑‑‑‑‑‑‑‑‑‑‑‑‑‑‑‑‑‑‑‑‑‑‑‑‑‑‑‑‑‑‑  >  NC_001604.1/12972‑13277`  
`‑‑‑‑‑‑‑‑‑‑‑‑‑‑‑‑‑‑‑‑‑‑‑‑‑‑‑‑‑‑‑‑‑‑‑‑‑‑‑‑‑‑‑‑‑‑‑‑‑‑‑‑‑‑‑‑‑‑‑‑‑‑‑‑‑‑‑‑‑‑‑‑‑‑‑‑‑‑‑‑‑‑‑‑‑‑‑‑‑‑‑‑‑‑‑‑‑‑‑‑‑‑‑‑‑‑‑‑‑‑‑‑‑‑‑‑‑‑‑‑‑‑‑‑‑‑‑‑‑‑‑‑‑‑‑‑‑‑‑‑‑‑‑‑‑‑‑‑‑‑‑‑‑‑‑‑‑‑‑‑‑‑‑‑‑‑‑‑‑‑‑‑‑‑‑‑‑‑‑‑‑‑‑‑‑‑‑‑‑‑‑‑‑‑‑‑‑‑‑‑‑‑‑‑‑‑‑‑‑‑‑‑‑‑‑‑‑‑‑‑‑‑‑‑‑‑‑‑‑‑‑‑‑‑‑‑‑‑‑‑‑‑‑‑‑‑‑‑‑‑‑‑‑‑‑‑‑‑‑‑‑‑‑‑‑‑‑‑‑‑‑‑‑‑‑‑‑‑‑‑‑‑‑‑‑‑‑‑‑‑‑‑‑‑‑‑‑‑‑ttcttgaCAGTTGTTTCATATGAAGAGATTGTTAAGTCACGATAATCAATAGGAGAAATCAATATGATCGTTTCTGACATCGAAGCTAACGCCCTCTTAGAGAGCGTCACTAAGTTCCACTGCGGGGTTATCTACGACTACTCCACCGCTGAGTACGTAAGCTACCGTCCGAGTGACTTCGGTGCGTATCTGGATGCGCTGGAAGCCGAGGTTGCACGAGGCGGTCTTATTGTGTTCCACAACGGTCACAAGTATGACGTTCCTGCATTGACCAAACTGGCAAAGTTGCAATTGAACCGAGAGTT  >  NC_001604.1/14297‑14594`  

                                                                                                                                                                                                                                                                                                                                                                                                                                                                                                                                                                                                                              
 `ACAAAGGTAAAGCACATGAGGAAGGTCGCCCCGTTTCTATTACTGACCTACGTGGTTCTGGCGCACTACGCCAACTATCTGATACTATTATTGCCCTTGGGCGTAATCAGCAAGGCGATATGCCTAACCTTGTCCTCGTTCGTATTCTCAAGTGCCGCTTTACTGGTGATACTGGTATCGCTGGCTACATGGAATACAACAAGGAAACCGGATGGCTTGAACCATCAAGTTACTCAGGGGAAGAAGAGTCACACTCAGAGTCAACAGACTGGTCCAACGACACTGACTTCTGACAGGATTC                                                                                                                                                                                                                                                                                                                <  1:6915/301‑1`  
      `GGTAAAGCACATGAGGAAGGTCGCCCCGTTTCTATTACTGACCTACGTGGTTCTGGCGCACTACGCCAACTATCTGATACTATTATTGCCCTTGAGCGTAATCAGCAAGGAGATATGCCTAACCTTGTCCTCGTTCGTATTCTCAAGTGCAGCTTTACTGGTGATACTGGTATCGCTGGCTACATGGAATACAACAAGGAAACCGGATGGCTTGAACCATCAAGTTACTCAGGGGAAGAAGAGTCACACTCAGAGTCAACAGACTGGTCCAACGACACTGACTTCTGACAGGATctgtctc                                                                                                                                                                                                                                                                                                           >  1:177458/1‑294`  
      `GGTAAAGCACATGAGGAAGGTCGCCCCGTTTATATTACTGACCTACGTGGTTCTGGCGCACTACGCCAACTATCTGATACTATTATTGCCCTTGAGCGTAATCAGCAAGGCGATATGCCTAACCTTGTCCTCGTTCGTATTCTCAAGTGCCGCTTTACTGGTGATACTGGTATCGCTGGCTACATGGAATACAACAAGGAAACCGGATGGCTTGAACCATCAAGTTACTCAGGGGAAGAAGAGTCACACGCAGAGTCAACAGACTGGTCCAACGACACTGACTTCTGAAAGGATTCTTGAC                                                                                                                                                                                                                                                                                                           >  1:419993/1‑301`  
           `AGCACATGAGGAAGGTCGCCCCGTTTCTATTACTGACCTACGTGGTTCTGGCGCACTACGCCAACTATCTGATACTATTATTGCCCTTGAGCGTAATCAGCAAGGCGATATGCCTAACCTTGTCCTCGTTCGTATTCTCAAGTGCCGCTTTACTGGTGATACTGGTATCGCTGGCTACATGGAATACAACAAGGAAACCGGATGGCTTGAACCATCAAGTTACTCAGGTGAAGAAGAGTCACACTCAGAGTCAACAGACTGGTCCAACGACACTGACTTCTGACAGGATTCTTGACAGTTG                                                                                                                                                                                                                                                                                                      <  1:706223/301‑1`  
                  `gatgtgtataagagacagTCTATTACTGACCTACGTGGTTCTGGCGCACTACGCCAACTATCTGATACTATTATTGCCCTTGAGCGTAATCAGCAAGGCGATATGCCTAACCTTGTCCTCGTTCGTATTCTCAAGTGCCGCTTTACTGGTGATACTGGTATCGCTTGCTACATGGAATACAACAAGGAAACCGGATGGCTTGAACCATCAAGTTACTCAGTGGAAGAAGAGTCACACTCAAAGTCAACAGACTGGTCCAACGACACTGACTTCTGACAGGATTCTTGACAGTTGTTTCATA                                                                                                                                                                                                                                                                                               <  1:211229/283‑1`  
                         `GTCGCCCCGTTTCTATTACTGACCTACGTGGTTCTGGCGCACTACGCCAACTATCTGATACTATTATTGCCCTTGAGCGTAATCAGCAAGGCGATATGCCTAACCTTGTCATCGTTCGTATTCTCAAGTGCCGCTTTACTGGTGATACTGGTATCGCTGGCTACATGGAATACAACAAGGAAACCGGATGGCTTGAACAATCAAGTTACTCAGGGGAAGAAGAGTCACACTCAGAGTCAACAGACTGGTCCAACGACACTGACTTCTGACAGGTTTCTTGACAGTTGTTTCATATGAAGAG                                                                                                                                                                                                                                                                                        >  1:157183/1‑301`  
                                `tgggctcggagatgtgtaaaagagacagGGCGCACTACGCCAACTATCTGATACTATTATTGCCCTTGAGCGTAATCAGCAAGGCGATATGCCTAACCTTGTCCTCGTTCGTATTCTCAAGTGCCGCTTTACTAGTGATACTGGTATCGCTGGCTACATGGAATACAACAAGGAAACCGGATGGCTTGAACCATCAAGTTACTCAGGGGAAGAAGAGTCACACTCAGAGTCAACAGACTGGTCCAACGACACTGACTTCTGACAGGATTCTTGACAGTTGTTTCATATGAAGAGATTGTTA                                                                                                                                                                                                                                                                                 <  1:63243/273‑1`  
                                 `GTTTCTATTACTGACCTACGTGGTTCTGGCGCACTACTCCAACTATCTGATACTATTATTGCCCTTGAGCGTAATCAGCAAGGCGATATGCCTAACCTTGTCCTCGTTCGTATTCTCAAGTGCCGCTTTACTGGTGATACTGGTATCGCTGGCTACATGGAATACAACAAGGAAACCGGATGGCTTGAACCATCAAGTTACTCAGTGGAAGAATAGTCACACTCATAGTCAACAGACTGGTCCAACGACACTGACTTCTGACAGGATTCTTGACAGTTGTTTCATATGAAGAGATTGTTAA                                                                                                                                                                                                                                                                                >  1:159501/1‑301`  
                                  `TTTCTATTACTGACCTACGTGGTTCTGGCGCACTACGCCAACTATCTGATACTATTATTGCCCTTGAGCGTAATCAGCAAGGCGATATGCCTAACCTTGTCCTCGTTCGTATTCTCAAGTGCCGCTTTACTGGTGATACTGGTATCGCTGGCTACATGGAATACAACAAGGAAACCGGATGGCTTGAACCATCAAGTTACTCAGGGGAAGAAGAGTCACACTCAGAGTCAACAGACTGGTCCAACGACACTGACTTCTGACAGGATTCTTGACAGTTGTTTCATATGAAGAGATTGTTAAG                                                                                                                                                                                                                                                                               >  1:393359/1‑301`  
                                  `TTTCTATTACTGACCTACGTGGTTCTGGCGCACTACGCCAACTATCTGATACTATTATTGCCCTTGAGCGTAATCAGCAAGGCGATATGCCTAACCTTGTCCTCGTTCGTATTCTCAAGTGCCGCTTTACTGGTGATACTGGTATCGCTGGCTACATGGAATACAACAAGGAAACCGGATGGCTTGAACCATCAAGTTACTCAGGGGAAGAAGAGTCACACTCAGAGTCAACAGACTGGTCCAACGACACTGACTTCTGACAGGATTCTTGACAGTTGTTTCATATGAAGAGATGGTTAAG                                                                                                                                                                                                                                                                               >  1:393333/1‑301`  
                                   `TTCTATTACTGACCTACGTGGTTCTGGCGCACTACGCCAACTATCTGATACTATTATTGCCCTTGAGCGTAATCAGCAAGGCGATATGCCTAACCTTGTCCTCGTTCGTATTCTCAAGTGCCGATTTACTGGTGATACTGGTATCGCTGGCTACATGGAATACAACAAGGAAACCGGATGGCTTGAACCATCAAGTTACTCAGGGGAAGAAGAGTCACACTCAGAGTCAACAGACTGGTCCAACGACACTGACTTCTGACAGGATTCTTGACAGTTGTTTCATATGAAGAGATTGTTAAGT                                                                                                                                                                                                                                                                              >  1:414579/1‑301`  
                                    `TCTATTACTGACCTACGTGGTTAGGGCGCACTACGCCAACTATCTGATACTAATATTGCCCTTGAGCGTAATCAGCAAGGCGATATGCCTAACCTTGTCCTCGTTCGTATTCTCAAGTGCCGCTTTACTGGTGATACTGGTATCGCTGGCTACATGGAATACAACAAGGAAACCGGATGGCTTGAACCATCAAGTTACTCAGGGGAAGAAGAGTCACACTCAGAGTCAACAGACTGGTCCAACGACACTGACTTCTGACAGGATTCTTGACAGTTGTTTCATATGAAGAGATTGTTAAGTC                                                                                                                                                                                                                                                                             <  1:346815/301‑1`  
                                       `ATTACTGACCTACGTGGTTCTGGCGCACTACGCCAACTATCTGATACTATTATTGCCCTTGAGCGTAATCAGCAAGGCGATATGCCTAACCTTGTCCTCGTTCGTATTCTCAAGTGCCGCTTTACTGGTGATACTGGTATCGCTGGCTACATGGAATACAACAAGGAAACCGGATGGCTTGAACCATCAAGTTACTCAGGGGAAGAAGAGTCACACTCAGAGTCAACAGACTGGTCCAACGACACTGACTTCTGACAGGATTCTTGACAGCTGTCTCTTATacacatctccgaacccacga                                                                                                                                                                                                                                                                          >  1:616585/1‑281`  
                                       `ATTACTGACCTACGTGGTTCTGGCGCACTACGCCAACTATCTGATACTATTATTGCCCTTGAGCGTAATCAGCAAGGCGATATGCCTAACCTTGTCCTCGTTCGTATTCTCAAGTGCCGCTTTACTGGTGATACTGGTATCGCTGGCTACATGGAATACAACAAGGAAACCGGATGGCTTGAACCATCAAGTTACTCAGGGGAAGAAGAGTCACACTAAGAGTCAACAGACTGGTCCAACGACACTGACTTCTGACAGGTTTCTTGACAGTTGTTTCATATGAAGAGATTGTTAAGTCACG                                                                                                                                                                                                                                                                          >  1:749291/1‑301`  
                                               `CCTACGTGGTTCTGGCGCACTACGCCAACTATCTGATACTATTATTGCCCTTGAGCGTAATCAGCAAGGCGATATGCCTAACCTTGTCCTCGTTCGTATTCTCAAGTGCCGCTTTACTGGTGATACTGGTATCTCTGGCTACATGGAATACAACAAGGAAACCGGATGGCTTGAACCATCAAGTTACTCAGGGTAAGAAGAGTCACACTCAGAGTCAACAGACTGGTCCAACGACACTGACTTCTGACAGGATTCTTGACAGTTGTTTCATATGAAGAGATTGTTAAGTCACGATAATCAA                                                                                                                                                                                                                                                                  >  1:375693/1‑301`  
                                                  `ACGTGGTTCTGGCGCACTACGCCAACTATCTGATACTATTATTGCCCTTGAGCGTAATCAGCAAGGCGATATGCCTAACCTTGTCCTCGTTCGTATTCTCAAGTGCCGCTTTACTGGTGATACTGGTATCGCTGGCTACATGGAATACAACAAGGAAACCGGATTGCTTGAACCATCAAGTTACTCAGGGGAAGAAGAGTCACACTCAGAGTCAACAGACTGGTCCAACGACACTGACTTCTGACAGGATTCTTGACAGTTGTTTCATATGAAGAGATTGTTAAGTCACtgtctcttataa                                                                                                                                                                                                                                                               >  1:278282/1‑289`  
                                                      `acagCTGGCGCACTACGCCAACTATCTGATACTATTATTGCCCTTGAGCGTAATCAGCAAGGCGATATGCCTAACCTTGTCCTCGTTCGTATTCTCAAGTGCCGCTTTACTGGTGATACTGGTATCGCTGGCTACATGGAATACAACAAGGAAACCGGATGGCTTGAACCATCAAGTTACTCAGGGGAAGAAGAGTCACTCTCAGAGTCAACAGACTGGTCCAACGACACTGACTTC‑‑‑‑‑‑‑‑‑‑‑‑TGACAGTTGTTTCATATGAAGAGATTGTTAAGTCACGATAATCAATAGGAGAAATCAATAGGAT                                                                                                                                                                                                                                               <  1:401140/297‑1`  
                                                                        `gtgtataagagacagATTATTTCCCTTGAGCGTAATCAGCAAGGCGATATGCCTAACCTTGTCCTCGTTCGTATTCTCAAGTGCCGCTTTACTGGTGATACTGGTATCGCTGGCTACATGGAATACAACAAGGAAACCGGATGGCTTGAACCATCAAGTTACTCAGGGGAAGAAGAGTCACACTCAGAGTCAACAGACTGGTCCAACGACACTGACTTCTGACAGGTTTCTTGACAGTTGTTTCATATGAAGAGATTGTTAAGTCACGATAATCAATAGGAGAAATCAATATGATCGTTTC                                                                                                                                                                                                                                         <  1:115183/286‑1`  
                                                                         `AACTATCTGATACTATTATTGCCCTTGAGCGTAATCAGCAAGGCGATATGCCTAACCTTGTCCTCGTTCGTATTCTCAAGTGCCGCTTTACTGGTGATACTTGTATCGCTGGCTACATGGAATACAACAAGGAAACCGGATGGCTTGAACCATCAAGTTACTCAGGGGAAGAAGAGTCACACTCAGAGTCAACAGACTGGTCTAACGACACTGACTTCTGACAGGATTCTTGACAGTTGTTTCATATGAAGAGATGGTTAAGGCACGATAATCAATAGGAGAAATCAATATGATCGTTTCT                                                                                                                                                                                                                                        >  1:466705/1‑301`  
                                                                         `AACTATCTGATACTATTATTGCCCTTGAGCGTAATCAGCAAGGCGATATGCCTAACCTTGTCCTCGTTCGTATTCTCAAGTGCCGCTTTACTGGTGATACTGGTATCGCTGGCTACATGGAATACAACAAGGAAACCGGATGGCTTGAACCATCAAGTTACTCAGGGGAAGAAGAGTCACACTCAGAGTCAACAGACTGGTCCAACGACACTGACTTCTGACAGGATTCTTGACAGTTGTTTCATATGAAGAGATTGTTAAGTCACGATAATCAATAGGAGAAAgcctttctcttatccac                                                                                                                                                                                                                                        >  1:750131/1‑284`  
                                                                             `tTCTGATACTATTATTGCCATTGAGCGTAATCAGCAAGGCGATATGCCTAACCTTGTCCTCGTTCGTATTCTCAAGTGACGCTTTACTGGTGATACTGGTATCGCTGGCTACATGGAATACAACAAGGAAACCGGATGGCTTGAACCATCAAGTTACTCAGGGGAAGAAGAGTCACACTCAGAGTCAACAGACTGGTCCAACGACACTGACTTCTGACAGGTTTCTTGACAGTTGTTTCATATGAAGAGATTGTTAAGTAACGATAATAAATAGGAGAAATAAATATGATCGTTTCTGACA                                                                                                                                                                                                                                    >  1:297306/2‑301`  
                                                                                     `CTATTATTGCCCTTTAGCGTAATCAGCAAGGCGATATGCCTAACCTTGTCCTCGTTCGTATTCTCAAGTGCCGCTTTACTGGTGATACTGGTATCGCTGGCTACATGGAATACAACAAGGAAACCGGATGGCTTGAACCATCAAGTTACTCAGGGGAAGAAGAGTCACACTCAGAGTCAACAGACTGGCCCAACGACACTGACTTC‑‑‑‑‑‑‑‑‑‑‑‑TGACAGTTGTTTCATATGAAGAGATTGTTAAGTCACGATAATCAATAGGAGAAATCAATATGATCGTTTCTGACATCGAAGCTAACGCCCTCTTA                                                                                                                                                                                                                >  1:334638/1‑301`  
                                                                                        `tttTTGCCCGTGAGCGTAATCAGCAAGAGAATATGCCTAACCTTGTCCTCGTTAGTATTCTCAAGTACCGCTTTACTGGAGATACTGGTATCGCTGGCTAGATGGAATACAACAAGGAAACCGGATGGCTTGAACCAGCCAGTTACTCAGGGGAAGAAGAGTCACACTCAGAGCAAACAGACTGGTCCAACGACACTGACTTC‑‑‑‑‑‑‑‑‑‑‑‑TGACAGTTGTTTCATATGAAGATATTGTTAAGTCACGATAATCAATAGGAGGAATCAATATGATCGTTTCTGACATCGAAGCTAACGCCCTCTTAGAG                                                                                                                                                                                                             <  1:89308/298‑1`  
                                                                                        `gggagatgtgtataagatacagGCAAGGCGATATGCCTAACCTTGTCCTCGTTCGTATTCTCAAGTGCCGCTTTACTGGTGATACTGGTATCGCTGGCTACATGGAATACAACAAGGAAACCGGATGGCTTGAACCATCAAGTTACTCAGGGGAAGAAGAGTCACACTAAGAGTCAACAGACTGGTCCAACGACACTGACTTC‑‑‑‑‑‑‑‑‑‑‑‑TGACAGTTGTTTCATATGAAGAGATTGTTAAGTCACGATAATCAATAGGAGAAATCAATATGATCGTTTCTGACATCGAAGCTAACGCCCTCTTAGAG                                                                                                                                                                                                             <  1:306952/279‑1`  
                                                                                        `TTATTGCCCTTGAGCGTAATCAGCAAGGCGATATGCCTAACCTTGTCCTCGTTCGTATTCTCAAGTGCCGCTTTACTGGTGATACTGGTATCGCTGGCTACATGGAATACAACAAGGAAACCGGATGGCTTGAACCATCAAGTTACTAAGGGGAAGAAGAGTCACACTCAGAGTCAACAGACTGGTCCAACGACACTGACTTC‑‑‑‑‑‑‑‑‑‑‑‑TGACAGTTGTTTCATATGAAGAGATTGTTAAGTCACGATAATCAATAGGAGAAATCAATATGATCGTTTCTGACATCGAAGCTAACGCCCTCTTAGAG                                                                                                                                                                                                             <  1:759505/301‑1`  
                                                                                          `aaattgggaaaaagagaagggaAAGGGGTTATGCTAAACCTTGCCCTCGTTGGTTTTCCAAAGGGCCGCTTACTTGGTGATACT‑GTATCGCTGGCTACATGGAATACAACAAGGAAACCGGATGGCTTGAACCATCAAGTTACTCATGGGAAGAAGAGTCACACTCAGAGTCAACAGACTGGTCCAACGACACTGACTTCTGACAGGTTTCTTGACAGTTGTTTCATATGAAGAGATTGTTAAGTCACGATAATCAATAGGAGAAATCAATATGATCGTTTCTGACATCGAAGCTAACGCC                                                                                                                                                                                                                      <  1:77894/279‑1`  
                                                                                              `CCCTTGAGCGTAATCAGCAAGGCGATATGCCTAACCTTGTCCTCGTTCGTATTCTCAAGTGCCGCTTTACTGGTGATACTGGTATCGCTGGCTACATGGAATACAACAAGGAAACCGGATGGCTTGAACCATCAAGTTACTCAGGGGAAGAAGAGTCACACTCAGAGTCAACAGACTGGTCCAACGACACTGACTTC‑‑‑‑‑‑‑‑‑‑‑‑TGACAGTTGTTTCATATGAAGAGATTGTTAAGTCATGATAATCAATATGAGAAATCAATATGATCGTTTCTGACATCGAAGCTAACTCCCTCTTAGAGAGCGTC                                                                                                                                                                                                       >  1:256912/1‑301`  
                                                                                               `CCTTGAGCGTAATCAGCAAGGCGATATGCCTAACCTTGTCCTCGTTCGTATTCTCAAGTGCCGCTTTACTGGTGATACTGGTATCGCTGGCTACATGGAATACAACAAGGAAACCGGATGGCTTGAACCATCAAGTTACTCAGGGGAAGAAGAGTCACACTCAGAGTCAACAGACTGGTCCAACGACACTGACTTCTGACAGGTTTCTTGACAGTTGTTTCATATGAAGAGATTGTTAAGTCACGATAATCAATAGGAGAAATCAATATGATCGTTTCTGACATCGAAGCTAACGCCCTCT                                                                                                                                                                                                                  >  1:368897/1‑301`  
                                                                                                  `TGAGCGTAATCAGCAAGGCGATATGCCTAACCTTGTCCTCGTTCGTATTCTCAAGTGCCGCTTTACTGGTGATACTGGTATCGCTGGCTACATGGAATACAACAAGGAAACCGGATGGCTTGAACCATCAAGTTACTCAGGGGAAGAAGAGTCACACTCAGAGTCAACAGACTGGTCCAACGACACTGACTTCTGACAGGTTTCTTGACAGTTGTTTCATATGAAGAGATTGTTAAGTCACGATAATCAATAGGAGAAATCAATATGATCGTTTCTGACATCGAAGCTAACGCCCTCTTAG                                                                                                                                                                                                               <  1:404701/301‑1`  
                                                                                                  `TGAGCGTAATCAGCAAGGCGATATGCCTAACCTTGTCCTCGTTCGTATTCTAAAGTGCCGCTTTACTGGTGATAATGGTATCGCTGGCTACATGGAATACAACAAGGAAACCGGATGGCTTGAACCATCAAGTTACTCAGGGGAAGAAGAGTCACACTCAGAGTCAACAGACTGGTCCAACGACACTGACTTCTGACAGGATTCTTGACAGTTGTTTCATATGAATAGATTGTTAAGTCACGATAATCAATAGGAGAAATCAATATGATCGTTTCTGACATCGAAGCTAACGCCCTCTTAG                                                                                                                                                                                                               <  1:385197/301‑1`  
                                                                                                    `ggaaatgtgtataagagtaatTGCCTATCCTTGTCCTCGTTCATATTCTCAAGTGCCGCTTTACTGGTGATACTGGTATCGCTGGCTACATGGAATACAACAAGGAAACCGGATGGCTTGAACCATCAAGTTACTCAGGGGAAGAAGAGTCACACTCAGAGTCAACAGACTGGTCCAACGACACTGACTTCTGACAGGATTCTTGACAGTTGTTTCATATGAAGAGATTGTTAAGTCACGATAATCAATAGGAGAAATCAATATGATCGTTTCTGACATCGAAGCTAACGCCCTCTTAGAG                                                                                                                                                                                                             <  1:514411/280‑1`  
                                                                                                    `gGCGTAATAAGAAAGGCGATATGCCTAACCTGGTCCTCGTTAGTATTCGCAAGTGCCGCTTTACTGGTGATACTGGTATCGCTGGCTACATGGAATACAACAAGGAAACCGGATGGCTGGAACCATCAAGTTACTCAGGGGAAGAAGAGTCACACTCAAAGTCAACAGACTGGTCCAACGACACTGACTTCTGACAGGATTCTTGACAGTTGTTTCATATGAAGAGATTGTTAAGTCACGATAATCAATAGGAGAAATCAATATGATCGTTTCTGACATCGAAGCTAACGCCCTATTAGAG                                                                                                                                                                                                             <  1:434324/300‑1`  
                                                                                                    `cggagatgtgtataagagacagGCCTAACCTTGTCCTCGTTCGTATTCTCAAGTGCCGCTTTACTGGTGATACTGGTATCGCTGGCTACATGGAATACAACAAGGAAACCGGATGGCTTGAACCATCAAGTTACTCAGTGGAAGAAGAGTCACACTCAGAGTCAACAGACTGGTCCAACGACACTGACTTCTGACAGGATTCTTGACAGTTGTTTCATATGAATAGATTGTTAAGTCACGATAATCAATAGGAGAAATCAATATGATCGTTTCTGACATCGAAGCTAACGCCCTCTTAGAG                                                                                                                                                                                                             <  1:661756/279‑1`  
                                                                                                    `cagGTAATCAGCAAGGCGATATGCCTAACCTTGTCTTCGTTCGTATTCTCAAGTGCCGCTTTACTGGTGATACTGGTATCGCTGGCTACATTGAATACAACAAGGAAACCGGATGGCTTGAACCATCAAGTTACTCAGGGGAAGAAGAGTCACACTCAGAGTCAACAGACTGGTCCAACGACACTGACTTCTGACAGGATTCTTGACAGTTGTTTCATATGAAGAGATTGTTAAGTCACGATAATCAATAGGAGAAATCAATATGATCGTTTCTGACATCGAAGCTAACGCACTCTTAGAG                                                                                                                                                                                                             <  1:253103/298‑1`  
                                                                                                    `AGCGTAATCAGCAAGGCTATATGCCTAACCTTGTCCTCGTTCGTATTCTCAAGTGCCGCTTTACTGGTGATACTGGTATCGCTGGCTACATGGAATACAACAAGGAAACCGGATGGCTTGAACCATCAAGTTACTCAGGGGAAGAAGAGTCACACTCAGAGTCAACAGACTGGTCCAACGACACTGACTTCTGACAGGATTCTTGACAGTTGTTTCATATGAAGAGATTGTTAAGTCACGATAATCAATAGGAGAAATCAATATGATCGTTTCTGATATCGAAGCTAACGCCCTCTTAGAG                                                                                                                                                                                                             <  1:228327/301‑1`  
                                                                                                    `AGCGTAATCAGCAAGGCGATATGCCTAACCTTGTCCTCGTTCGTATTCTCAAGTGCCGCTTTACTGGTGATACTGGTATCGCTGGCTACATGGAATACAACAAGGAAACTGGATGGCTTGAACCATCAAGTTACTCAGGGGAAGAAGAGTCACACTCAGAGTCAACAGACTGGTCCAACGACACTGACTTCTGACAGGATTCTTGACAGTTGTTTCATATGAAGAGATTGATAAGTCACGATAATCAATAGGAGAAATCAATATGATCGTTTCTGACATCGAAGCTAACGCCCTCTTAGAG                                                                                                                                                                                                             <  1:816039/301‑1`  
                                                                                                    `AGCGTAATCAGCAAGGCGATATGCCTAACCTTGTCCTCGTTCGTATTCTCAAGTGCCGCTTTACTGGTGATACTGGTATCGCTGGCTACATGGAATACAACAAGGAAACCGGATGGCTTGAACCATCAAGTTACTCAGGGGAAGAAGAGTCACACTCAGAGTCAACAGACTGGTCCAACGACACTGACTTCTGACAGGATTCTTGACAGTTGTTTCATATGAAGAGATTGTTAAGTCACGATAATCAATAGGAGAAATCAATATGATCGTTTCTGACATCGAAGCTAACGCCCTCTTAGAG                                                                                                                                                                                                             <  1:23827/301‑1`  
                                                                                                    `AGCGTAATCAGCAAGGCGATATGCCTAACCTTGTCCTCGTTCGTATTCTCAAGTGCCGATTTACTGGTGATACTGGTATCGCTGGCTACATGGAATACAACAAGGAAACCGGATGGCTTGAACCATCAAGTTACTCAGGGGAAGAAGAGTCACACTCAGAGTCAACAGACTGGTCCAACGACACTGACTTCTGACAGGATTCTTGACAGTTGTTTCATATGAAGAGATTGTTAAGTCACGATAATCAATTGGAGAAATCAATATGATCGTTTCTGACATCGAAGCTAACGCACTCTTAGAG                                                                                                                                                                                                             <  1:122802/301‑1`  
                                                                                                      `CGTAATCAGCAAGGCGATATGCCTAACCTTGTCCTCGTTCGTATTCTCAAGTGCCGCTTTACTGGTGATACTGGTATCGCTGGCTACATGGAATACAACAAGGAAACCGGATGGCTTGAACCATCAAGTTACTCAGGGGAAGAAGAGTCACACTCAGAGTCAACAGACTGGTCCAAAGACACTGACTTCTGACAGGATTCTTGACAGTTGTTTCATATGAAGAGATTGTTAAGTCACGATAATCAATAGGAGAAATCAATATGATCGTTTCTGACATCGAAGCTAACGCCCTCTTAGAGAG                                                                                                                                                                                                           <  1:151212/301‑1`  
                                                                                                               `gtgtataagagacagAACCTTGTCCTCGTTCGGATTCTCAAGTGCCGATTTACTGGTTATACTGGTATCGCTGGCTACATGGAATACAACAAGGAAACCGGATGGCTTGAACCATCAAGTTACTCAGGGGAAGAAGAGTCACACTCAGAGTCAACAGACTGGTCCAACGACACTGACTTC‑‑‑‑‑‑‑‑‑‑‑‑TGACAGTTGTTTCATATGAAGAGATTGTTAAGTCACGATAATCAATAGGAGAAATCAATATGATCGTTTCTGACATCGAAGCTAACGCCCTCTTAGAGAGCGTCACTAAGTTCCACTGCGG                                                                                                                                                                                      <  1:770647/286‑1`  
                                                                                                                 `AGGCGATATGCCTAACCTTGTCCTCGTTCGTATTCTCAAGTGCCGCTTTACTGGTGATACTGGTATTGCTGGCTACATGGAATACAACAAGGAAACCGGATGGCTTGAACCATCAAGTTACTCAGGGGAAGAAGAGTCACACTCAGGGTCAACAGACTGGTCCAACGACACTGACTTCTGACAGGATTCTTGACAGTTGTTTCATATGAAGAGATTGTTAAGTCACGATAATCAATAGGAGAAATCAATATGATCGTTTCTGACATCGAAGCTAACGCCCTCTTATAGAGCGTCACTAAGT                                                                                                                                                                                                <  1:581216/301‑1`  
                                                                                                                 `AGGCGATATGCCTAACCTTGTCCTCGTTCGTATTCTCAAGTGCAGCTTTACTGGTGATACTGGTATCGCTGGCTACATGGAATACAACAAGGAAACCGGATGGCTTGAACCATCAAGTTACTCAGGGGAAGAAGAGTCACACTCAGAGTCAACAGACTGGTCCAACGACACTGACTTCTGACAGGATTCTTGACAGTTGTTTCATATGAAGAGATTGTTAAGTCACGATAATCAATAGGAGAAATCAATATGATCGTTTCTGACATCGAAGCTAACGCCCTCTTAGAGAGCGTCACTAAGT                                                                                                                                                                                                <  1:743682/301‑1`  
                                                                                                                      `cagTGCCTAACCTTGTCCTCGTTCGTATTCTCAAGTGCCGCTTTACTTGTGATACTGGTATCGCTGGCTACATGGAATACAACAAGGAAACCGGATGGCTTGAACCATCAAGTTACTCATGGGAAGAAGAGTCACACTCAGAGTCAACAGACTGGTCCAACGACACTGACTTCTGACAGGATTCTTGACAGTTGTTTCATATGAAGAGATTGTTAAGTCACGATAATCAATAGGAGAAATCAATATGATCGTTTCTGACATCGAAGCTAACGCCCTCTTAGAGAGCGTCACTAAGTTCCAC                                                                                                                                                                                           >  1:542253/4‑301`  
                                                                                                                        `ATGCCTAACCTTGTCCTCGTTCGTATTCTCAAGTGCCGCTTTACTGGTGATACTGGTATCGCTGGCTACATGGAATACAACAAGGAAACCGGATGGCTTGAACCATCAAGTTACTCAGGGGAAGAAGAGTCACACTCAGAGTCAACAGACTGGTCCAACGACACTGACTTCTTACAGGATTCTTGACAGTTGTTTCATATGAAGAGATTGTTAAGTCACGATAATCAATAGGAGAAATCAATATGATCGTTTCTGACATCGAAGCTAACGCCCTCTTAGAGAGCGTCACTAAGTTCCACTG                                                                                                                                                                                         >  1:413804/1‑301`  
                                                                                                                          `GCCTAACCTTGTCCTCGTTCGTATTCTCAAGTGCCGCTTTACTGGTGATACTGGTATCGCTGGCTACATGGAATACAACAAGGAAACCGGATGGCTTGAACCATCAAGTTACTCAGGGGAAGAAGAGTCACACTCAGAGTCAACAGACTGGTCCAACGACACTGACTTCTGACAGGATTCTTGACAGTTGTTTCATATGAAGAGATTGTTAAGTCACGATAATCAATAGGAGAAATCAATATGATCGTTTCTGACATCGAAGCTAACGCCCTCTTAGAGAGCGTCACTAAGTTCCACTGCG                                                                                                                                                                                       >  1:752554/1‑301`  
                                                                                                                          `GCCTAACCTTGTCCTCGTTCGTATTCTCAAGTGCCGCTTTACTGGTGATACTGGTATCGCTGGCTACATGGAATACAACAAGGAAACCGGATGGCTTAAACCATCCAGTTACTCAGGTGAAGAAGAGTCACACTCAGAGTCAACAGACTGGTCCAACGACACTGACTTCTGACAGGATTCTTGACAGTTGTTTCATATGAAGAGATTGTTAAGTCACGATAATCAATAGGAGAAATCAATATGATCGTTTCTGACATCGAAGCTAACGCCCTCTTAGAGCGCGTCACTAAGTctgtctctt                                                                                                                                                                                       >  1:700292/1‑292`  
                                                                                                                          `GCCTAACCTTGTCCTCGTTCGTATTCTCAAGTGCCGCTTTACTGGTGATACTGGTATCGATGGCTACATGGAATACAACAAGGAAACCGGATGGCTTGAACCATCAAGTTACTCAGGGGAAGAAGAGTCACACTCAGAGTCAACAGACTGGTCCAACGACACTGACTTCTGACAGGATTCTTGACAGTTGTTTCATATGAAGAGATTGTTAAGTCACGATAATCAATAGGAGAAATCAATATGATCGTTTCTGACATCGAAGCTAACGCCCTCTTAGAGctgtctcttatacacatctccg                                                                                                                                                                                       >  1:455247/1‑279`  
                                                                                                                                       `CTCGTTCGTATTCTCAAGTGCCGCTTGACTGGTGATACTGGTATCGCTGGCTACATGGAATACAACAAGGAAACCGGATGGCTTGAACCATCAAGTTACTCAGGGGAAGAAGAGTCACACTCAGAGTCAACAGACTGGTCCAACGACACTGACTTC‑‑‑‑‑‑‑‑‑‑‑‑TGACAGTTGTTTCATATGAAGAGATTGTTAAGTCACGATAATCAATAGGAGAAATCAATATGATCGTTTCTGACATCGAAGCTAACGCCCTCTTAGAGAGCGTCACTAAGTTCCACTGCGGGGTTATCTACGACTACTCCACCGC                                                                                                                                                              <  1:502079/301‑1`  
                                                                                                                                       `CTCGTTCGTATTCCCAAGTGCCGCTTTACTGGTGATACTGGTATCGCTGGCTACATGGAATACAACAAGGAAACCGGATGGCTTGAACCATCAAGTTACTCAGGGGAAGAAGAGTCACACTCAGAGTCAACAGACTGGTCCAACGACACTGACTTC‑‑‑‑‑‑‑‑‑‑‑‑TGACAGTTGTTTCATATGAAGAGATTGTTAAGTCACGATAATCAATAGGATAAATCAATATGATCGTTTCTGACATCGAAGCTAACGCCCTCTTAGAGAGCGTCACTAAGTTCCACTGCGGGGTTATCTACGACTACTCCACCGC                                                                                                                                                              <  1:316665/301‑1`  
                                                                                                                                        `agGTTCGTATTCTCAAGTGCCGCTTTACTGGTGATACTGGTATCGCTGGCTACATGGAATACAACAAGGAAACCGGATGGCTTGAACCATCAAGTTACTCAGGGGAAGAAGAGTCACACTCAGAGTCAACAGACTGGTCCAACGACACTGACTTCTGACAGGATTCTTGACAGTTGTTTCATATGAAGAGATTGTTAAGTCACGATAATCAATAGGAGAAATCAATATGATCGTTTCTGACATCGAAGCTAACGCCCTCTTAGAGAGCGTCACTAAGTTCCACTGCGGGGTTATCTACGAC                                                                                                                                                                         <  1:360646/299‑1`  
                                                                                                                                        `TCGTTCGTATTCTCAAGTGCCGCTTTACTGGTGATACTGGTATCGCTGGATACATGGAATACAACAAGGAAACCGGATGGCTTGGACCATCAAGTTACTCAGGGGAAGAAGAGTCACACTCAGAATCAACAGACTGGTCCAACGACACTGACTTCTGACAAGATTCTTGACAGTTGTTTCATATGAAGAGATTGTTAAGTCAAGATAATCAATAGGAGAAATCAATATGATCGTTTCTGACATCGAAGCTAAAGCCCTCTTAGAGAGCGTCACTAAGTTCCACTGCGGGGTTATCTACGAC                                                                                                                                                                         <  1:229849/301‑1`  
                                                                                                                                         `CGTTCGTATTCTCAAGTGCCGCTTTACTGGTGATACTGGTATCGCTGGCTACATGGAATACAACAAGGAAACCGGATGGCTTGAACCATCAAGTTACTCAGGGGAAGAAGAGTCACACTCAGAGTCAACAGACTGGTCCAACGACACTGACTTCTGACAGGATTCTTGACAGTTGTTTCATATGAAGAGATTGTTAAGTCACGATAATCAATAGGAGAAATCAATATGATCGTTTCTGACATCGAAGCTAACGCCCTCTTAGAGAGCGTCACTAAGTTCCACTGCGGGGTTATCTACGACT                                                                                                                                                                        >  1:38169/1‑301`  
                                                                                                                                           `TTCGTATTCTCAAGTGCCGCTTTACTGGTGATACTGGTATCGCTGGCTACATGGAATACAACAAGGAAACCGGATGGCTTGAACCATCAAGTTACTCAGGGGAAGAAGAGTCACACTCAGAGTCAACAGACTGGTCCAACGACACTGACTTCTGACAGGTTTCTTGACAGTTGTTTCATATGAAGAGATTGTTAAGTCACGATAATCAATAGGAGAAATCAATATGATCGTTTCTGACATCGAAGCTAACGCCCTCTTAGAGAGCGTAACTAAGTTCCACTGCGGGGTTATCTACGACTAC                                                                                                                                                                      >  1:480598/1‑301`  
                                                                                                                                           `TTCGTATTCTCAAGTGCCGCTTTACTGGTGATACTGGTATCGCTGGCTACATGGAATACAACAAGGAAACCGGATGGCTTGAACCATCAAGTTACTCAGGGGAAGAAGAGTCACACTCAGAGTCAACAGACTGGTCCAACGACACTGACTTCTGACAGGATTCTTGACAGTTGTTTCATATGAAGAGATTGTTAAGTCACGATAATCAATAGGAGAAATCAATATGATCGTTTCTGACATCGAAGATAACGCCCTCTTAGAGAGCGTCACTActgtctcttatacacatctccgagcccag                                                                                                                                                                      >  1:774758/1‑272`  
                                                                                                                                             `ggctcggagatgtgtataagagacaGTGATACTGGTATCGCTGGCTACATGGAATACAACAAGGAAACCGGATGGCTTGAACCATCAAGTTACTCAGGGGAAGAAGAGTCACACTCAGAGTCAACAGACTGGTCCAACGACACTGACTTCTGACAGGATTCTTGACAGTTGTTTCATATGAAGAGATTGTTAAGTCACGATAATCAATAGGAGAAATCAATATGATCGTTTCTGACATCGAAGCTAACGCCCTCTTAGAGAGCGTCACTAAGTTCCACTGCGGGGTTATCTACGACTACTC                                                                                                                                                                    <  1:101125/276‑1`  
                                                                                                                                                   `tcggagatgtgtataagagaaagTACTGGTATCGCTGGCTACATGGAATACAACAAGGAAACCGGATGGCTTGAACCATCAAGTTACTCAGGGGAAGAAGAGTCACACTCAGAGTCAACAGACTGGTCCAACGACACTGACTTC‑‑‑‑‑‑‑‑‑‑‑‑TGACAGTTGTTTCATATGAAGAGATTGTTAAGTCACGATAATCAATAGGAGAAATCAATATGATCGTTTCTGACATCGAAGCTAACGCCCTCTTAGAGAGCGTCACTAAGTTCCACTGCGGGGTTATCTACGACTACTCCACCGCTGAGTACGTAAG                                                                                                                                                  <  1:677385/278‑1`  
                                                                                                                                                   `CTCAAGTGCCGCTTTACTGGTGATACTGGTATCGCTGGCTACATGGAATACAACAAGGAAACCGGATGGCTTGAACCATCAAGTTACTCAGGGGAAGAAGAGTCACACTCAGAGTCAACAGACTGGTCCAACGACACTGACTTCTGACAGGATTCTTGACAGTTGTTTCATATGAAGAGATTGTTAAGTCACGATAATCAATAGGAGAAATCAATATGATCGTTTCTGACATCGAAGCTAACGCCCTCTTAGAGAGCGTCACTAAGTTCCACTGCTGGGTTATCTACGACTACTCCACCGC                                                                                                                                                              >  1:588617/1‑301`  
                                                                                                                                                       `AGTGCCGCTTTACTGGTGATACTGGTATCGCTGGCTACATGGAATACAACAAGGAAACCGGATGGCTTGAACCATCAAGTTACTCAGGGGAAGAAGAGTCACACTCAGAGTCAACAGACTGGTCCAACGACACTGACTTCTGACAGGATTCTTGACAGTTGTTTCATATGAAGAGATTGTTAAGTCACGATAATCAATAGGAGAAATCAATATGATCGTTTCTGACATCGAAGCTAACGCCCTCTTAGAGAGCGTCACTAAGTTCCACTGCGGGGTTATCTACGACTACTCCACCACTGAG                                                                                                                                                          <  1:240492/301‑1`  
                                                                                                                                                               `ataagagacagTACTGGTATCGCTGGCTACATGGAATACAACAAGGAAACCGGATGGCTTGAACCATCAAGTTACCCAGGGGAAGAAGAGTCACTCTCAGAGTCAACAGACTGGTCCAACGACACTGACTTCTGACAGGATTCTTGACAGTTGTTTCATATGAAGAGATTGTTAAGTCACGATAATCAATAGGAGAAATCAATATGATCGTTTCTGACATCGAAGCTAACGCCCTCTTAGAGAGCGTCACTAAGTTCCACTGCGGGGTTATCTACGACTACTCCACAGCTGAGTACGTAAG                                                                                                                                                  <  1:264876/290‑1`  
                                                                                                                                                               `TTTACTGGTGATACTGGTATCGCTGGCTACATGGAATACAACAAGGAAACCAGATGGCTTGAACCATCAAGTTACTAAGGGGAAGAAGAGTCACACTCAGAGTCAACAGACTGGTCCAACGACACTGACTT‑‑‑‑‑‑‑‑‑‑‑‑TTGACAGTTGTTTCATATGAAGAGATTGTTAAGTCACGATAATCAATAGGAGAAATCAATATGATCGTTTCTGACATCGAAGCTAACGCCCTCTTAGAGAGCGTCACTAAGTTCCACTGCGGAGTTATCTACGACTACTCCACCGCTGAGTACGTAAGCTACCGTCCGAG                                                                                                                                      <  1:524946/301‑1`  
                                                                                                                                                                `ttacgaGTGATACTGGTATCGCTGGCTACAGGCAATACAACAAGGAAACTGGATGGCTTGAACCATCAAGTTACTCAGGGGAAGAAGAGTCACACTCAGAGTCAACAGACTTGTCCAACGACACTGACTTCTGACAGGATTCTTGACAGTTGTTTCATATGAAGAGATTGTTAAGTCACGATAATCAATAGGAGCAATCAATATGATCGTATCTTACATCGAAGCTAACGCCCTCTTAGAGAGCGGCACGAAGTTCCACTGCGGGGTTATCTACGACTACTCCAGCGCTGAGTACGTAAGC                                                                                                                                                 <  1:267298/295‑1`  
                                                                                                                                                                 `TACTGGTGATACTGGTATCGCTGGCTACATGTAATACAACAAGGAAACCGAATGGCTTGAACCATCAAGTTACTCAGGGGAAGAAGAGTCACACTCAGAGTCAACAGACTGGTCCAACGACACTGACTTCTGACAGGTTTCTTGACAGTTGTTTCATATGAAGAGATTGTTAAGTCACGATAATCAATAGTAGAAATCAATATGATCGTTTCTGACATCGAAGCTAACGCCCTCTTAGAGAGCGTCACTAAGTTCCACTGCGGGGTTATCTACGACTACTCCACCGCTGAGTACGTAAGCT                                                                                                                                                <  1:797578/301‑1`  
                                                                                                                                                                  `ACTGGTGATACTGGTATCGCTGGCTACATGGAATACAACAAGGAAACCGGATGGCTTGAACCATCAAGTTACTCAGGGGAAGAAGAGTCACACTCAGAGTCAACAGACTGGTCCAACGACACTGACTTCTGACAGGATTCTTGACAGTTGTTTCATATGAAGAGATTGTTAAGTCACGATAATCAATAGGAGAAATCAATATGATCGTTTCTGACATCGAAGCTAACGCCCTCTTAGAGAGCGTCACTAAGTTCCACTGCGGGGTTATCTACGACTACTCCACCGCTGAGTAAGTAAGCTA                                                                                                                                               >  1:163486/1‑301`  
                                                                                                                                                                   `CTGGTGATACTGGTATCGCTGGCTACATGGAATACAACAAGGAAACCGGATGGCTTGAACCATCAAGTTACTCAGGGGAAGAAGAGTCACACTCAGAGTCACCAGACTGGTCCAACGACACTGACTTCTGACAGGATTCTTGACAGTTGTTTCATATGAAGAGATTGTTAAGTCACGATAATCAATAGGAGAAATCAATATGATCGTTTCTGACATCGAAGCTAACGCCCTCTTAGAGAGCGTCACTAAGTTCCACTGCAGGGTTATCTACGCCTACTCCACCGCTGAGTACGcacggtcc                                                                                                                                              >  1:425812/1‑293`  
                                                                                                                                                                   `CTGGTGATACTGGTATCGCTGGCTACATGGAATACAACAAGGAAACCGGATGGCTTGAACCATCAAGTTACTCAGGGGAAGAAGAGTCACACTCAGAGTCAACAGACTGGTCCAACGACACTGACTTC‑‑‑‑‑‑‑‑‑‑‑‑TGACAGTTGTTTCATATGAAGAGATTGTTAAGTCACGATAATCAATAGGAGAAATCAATATGATCGTTTCTGACATCGAAGCTAACGCCCTCTTAGAGAGCGTCACTAAGTTCCACTGCGGGGTTATCTACGACTACTACACCGCTGAGTACGTAAGCTACAGTCCGAGTGAC                                                                                                                                  >  1:493897/1‑301`  
                                                                                                                                                                         `ATACTGGTATCGCTGGCTACATGGAATACAACAAGGAAACCGGATGGCTTGAACCATCAAGTTACTCAGGGGAAGAAGAGTCACACTCAGAGTCAACAGACTGGTCCAACGACACTGACTTCTGACAGTATTCTTGACAGTTGTTTCATATGAATAGATTGTTAAGTCACGATAATCAATAGGAGAAATCAATATGATCGTTTCTGACATCGAAGCTAACGCCCTCTTAGAGAGCGTCACTAAGTTCCACTGCGGGGTTATCTACGACTACTCCACCGCTGAGTACGTAAGCTACCGTCtg                                                                                                                                        >  1:289552/1‑299`  
                                                                                                                                                                                    `GCTGGCTACATGGAATACAACAAGGAAACCGGATGGCTTGAACCATCAAGTTACTCAGGGGAAGAAGAGTCACACTCAGAGTCAACAGACTGGTCCAACGACACTGACTTCTGACAGGATTCTTGACAGTTGTTTCATATGAAGAGATTGTTAAGTCACGATAATCAATAGGAGAAATCAATATGATCGTTTCTGACATCGAAGCTAACGCCCTCTTAGAGAGCGTCACTAAGTTCCACTGCGGGGTTATCTACGACTACTCCACCGCTGAGTACGTAAGCTACCGTCAGAGTGACTTCGG                                                                                                                             <  1:111998/301‑1`  
                                                                                                                                                                                    `GCTGGCTACATGGAATACAACAAGGAAACCGGATGGCTTGAACCATCAAGTTACTCAGGGGAAGAAGAGTCACACTCAGAGTCAACAGACTGGTCCAACGACACTGACTTCTGACAGGATTCTTGACAGTTGTTTCATATGAAGAGATTGTTAAGTCACGATAATCAATAGGAGAAATCAATATGATCGTTTCTGACATCGAAGCTAACGCCCTCTTAGAGAGCGTCACTAAGTTCCACTGCGGGGTTATCTACAACTACTCCACCGCTGAGTACGTAAGCTACCGTCCGAGTGACTTCGG                                                                                                                             <  1:564631/301‑1`  
                                                                                                                                                                                    `GCTGGCTAAATGGAATACAAAAAGGAAACCGGATGGCTTGAACCATCAAGTTACTCAGGGGAAGAAGAGTCACACTCAGAGTCAACAGACTGGTCCAACGACACTGACTTC‑‑‑‑‑‑‑‑‑‑‑‑TGACAGTTGTTTCATATGAAGAGATTGTTAAGTCACGATAATCAATAGGAGAAATCAATATGATCGTTTCTGACATCGAAGCTAACGCCCTCTTAGAGAGCGTCACTAAGTTCCACTGCGGGGTTATCTACGACTACTCCACCGCTGAGTACGTAAGCTACCGTCCGAGTGACTTCGGTGCGTATCTGGA                                                                                                                 <  1:182938/301‑1`  
                                                                                                                                                                                     `gtgggctcggagatgtgtataagagacagGGATGGCTTGAACCATCAAGTTACTCAGGGGAAGAAGAGTCACACTCAGAGTCAACAGACTGGTCCAACGACACTGACTTC‑‑‑‑‑‑‑‑‑‑‑‑TGACAGTTGTTTCATATGAAGAGATTGTTAAGTCACGATAATCAATAGGAGAAATCAATATGATCGTTTCTGACATCGAAGCTAACGCCCTCTTAGAGAGCGTCACTAAGTTCCACTGCGGGGTTATCTACGACTACTACACCGCTGAGTACGTAAGCTACCGTCCGAGTGACTTCGGTGCGTATCTGGAT                                                                                                                <  1:138329/272‑1`  
                                                                                                                                                                                       `GGCTACATGGAATACAACAAGGAAACCGGATGGCTTGAACCATCAAGTTACTCATGGGAGGAAGAGTCACACTCAGAGTCAACATACTGGTCCAACGACACTGACTTCTGACAGGATTCTTGACAGTTGTTTCATATGAAGAGATTGTTAAGTCACGATAATCAATAGTAGAAATCAATATGATCGTTTCTGACATCGAAGCTAACGCCCTCTTAGAGAGCGTCACTAAGTTCCACTGCGGGGTTATCTACGACTACTCCACCGCTGAGTACGTAAGCTACCGTCCGAGTGACTTCGGctg                                                                                                                          >  1:527656/1‑298`  
                                                                                                                                                                                           `ACATGGAATACAACAAGGAAACCGGATGGCTTGAACCATCAAGTTACTCAGGGGAAGAAAAGTCACACTCAGAGTCAACAGACTGGTCCAACGACACTGACTTCTGACAGGATTCTTGACAGTTGTTTCATATGAAGAGATTGTTAAGTCACGATAATCAATAGGAGAAATCAATATGATCGTTTCTGACATCGAAGCTAACGCCCTCTTAGAGAGCGTCACTAAGTTCCACTGCGGGGTTATCTACGACTACTCAACCGCTGAGTACGTAAGCTACCGTCCGAGTGACTTCGGTGCGTAT                                                                                                                      >  1:194034/1‑301`  
                                                                                                                                                                                            `ttgtataagagACAGGGAAACCGGATGGCTTGAACCATCAAGTTACTAAGGGGAAGAAGAGTCACACTCAGAGTCAACAGACTGGTCCAACGACACTGACTTCTGACAGGATTCTTGACAGTTGTTTCATATGAAGAGATTGTTAAGTCACGATAATCAATAGGAGAAATCAATATGATCGTTTCTGACATCGAAGCTAACGCCCTCTTAGAGAGCGTCACTAAGTACCACTGCGGGGTTATCTACGACTACTCCACCGCTGAGTACGTAAGCTACCGTCCGAGTGACTTCGGTGCGTATC                                                                                                                     <  1:168311/290‑1`  
                                                                                                                                                                                                `GAATACAACAAGGAAACCGGATTGCTTGAACCATCAAGTTACTCAGTGGAAGAAGAGTCACACTCAGAGTCAACAGACTGGTCCAACGACACTGACTTCTGACAGGATTCTTGACAGTTGTTTCATATGAAGAGATTGTTAAGTCACGATAATCAATAGGAGAAATCAATATGATCGTTTCTGACATCGAAGCTAACGCCCTCTTAGAGAGCGTCACTAAGTTCCACTGCGGGGTTATCTACGACTACTCCACCGCTGAGTACGTAAGCTACCGTCCGAGTGACTTCGGTGCGTATCTGGt                                                                                                                 >  1:20418/1‑300`  
                                                                                                                                                                                                `GAATACAACAAGGAAACCGGATGGCTTGAACCATCAAGTTACTCAGGGGAAGAAGAGTCACACTCAGAGTCAACAGACTGGTCCAACGACACTGACTTCTGACAGGATTCTTGACAGTTGTTTCATATGAAGAGATTGTTAAGTCACGATAATCAATAGGAGAAATCAATATGATCGTTTCTGACATCGAAGCTAACGCCCTCTTAGAGAGCGTCACTAAGTTCCACTGCGGGGTTATCTACGACTACTCCACCGCTGAGTACGTAAGCTACCGTCCGAGTGACTTCGGTGCGTATCTGGg                                                                                                                 >  1:21464/1‑300`  
                                                                                                                                                                                                `GAATACAACAAGGAAACCGGATGGCTTGAACCATCAAGTTACTCAGGGGAAGAAGAGTCACACCCAGAGTCAACAGACTGGTACAACGACACTGACTTCTGACAGGATTCTTGACAGTTGTTTCATATGAAGAGATTGTTAAGTCACGATAATCAATAGGAGAAATCACTATGATCGTTTCTGACATCGAAGCTAACGCCCTCTTATAGAGCGTCACTAAGTTCCACTGCGGGGTTATCTACGACTACTCCACCGCTGCGTACGTACGCTCCCGTCCGAGTGCCTTCGGTGCGTATCTtgt                                                                                                                 >  1:41797/1‑298`  
                                                                                                                                                                                                    `ACAACAAGGAAACCGGATGGCTTGAACCATCAAGTTACTCAGGGGAAGAAGAGTCACACTCAGAGTCAACAGACTGGTCCAACGACACTGACTTCTGACAGGATTCTTGACAGTTGTTTCATATGAAGAGATTGTTAAGTCACGATAATCAATAGGAGAAATCAATATGATCGTTTCTGACATCGAAGCTAACGCCCTCTTAGAGAGCGTCACTAAGTTCCACTGCGGGGTTATCTACGACTACTCCACCGCTGAGTACGTAAGCTACCGTCCGAGTGACTTCAGTGCGTATCTGGATGCG                                                                                                             >  1:504607/1‑301`  
                                                                                                                                                                                                     `ataagagacagCCGGATGGCTTGAACCATCAAGTTACTCAGGGGAATAAGAGTCACACTCAGAGTCAACAGACTGGTCCAACGACACTGACTTCTGACAGGATTCTTGACAGTTGTTTCATATGAAGAGATTGTTAAGTCACGATAATCAATATGATAAATCAATATGATCGTTTCTGACATCGAAGCTAACGCCCTCTTAGAGAGCGTCACTAAGTTCCACTGCGGGGTTATCTACGACTACTCCACCGCTGAGTACGTAAGCTACCGTCCGAGTGACTTCGGTGCGTATCTGGATGCGC                                                                                                            <  1:329818/290‑1`  
                                                                                                                                                                                                                `ctcggagatgtgtataagagacagACTCAGGGGAAGAAGCGTCACACTCATAGTCAACAGACTGGTCCAACGACACTGACTTCTGACAGTATTCTTGACAGTTGTTTCATATGAAGAGATTGTTAAGTCACGATAATCAATAGGAGAAATCAATATGATCGTTTCTGACATCGAAGCTAACGCCCTCTTAGAGAGCGTCACTAAGTTCCACTGCGGGGTTATCTACGACTACTCCACCGCTGAGTACGTAAGCTACCGTCCGAGTGACTTCGGTGCGTATCTGGATGCGCTGGAAGCCGAG                                                                                                 <  1:448746/277‑1`  
                                                                                                                                                                                                                       `GCTTGAACCATCAAGTTACTCAGGGGAAGAAGAGTCACACTCAGAGTCAACAGACTGGTCCAACGACACTGACTTCTGACAGGATTCTTGACAGTTGTTTCATATGAAGAGATTGTTAAGTCACGATAATCAATAGGAGAAATCAATATGATCGTTTCTGATATCGAAGCTAACGCCCTCTTAGAGAGCGTCACTAAGTTCCACTGCGGGGTTATCTACGACTACTCCACCGCTGAGTACGTAAGCTACCGTCCGAGTGACTTCGGTGCGTATCTGGATGCGCTGGAAGCCGAGGTTGCAC                                                                                          <  1:56972/301‑1`  
                                                                                                                                                                                                                              `CCATCAAGTTACTCAGGGGAAGAAGAGTCACACTCAGAGTCAACAGACTGGTCCAACGACACTGACTTCTGACAGGATTCTTGACAGTTGTTTCATATGAAGAGATTGTTAAGTCACGATAATCAATAGGAGAAATCAATATGATCGTTTCTGACATCGAAGCTAACGCCCTCTTAGAGAGCGTCACTAAGTTCCACTGCGGGGTTATCTACGACTACTCCACCGCTGAGTACGTAAGCTACCGTCCGAGTGACTTCGGTGCGTATCTGGAGGCGCTGGAAGCCGAGGTTGCACGAGGCGG                                                                                   >  1:235674/1‑301`  
                                                                                                                                                                                                                               `CATCAAGTTACTCAGTGGAAGAATAGTCACACTCAGAGTCAACAGACTGGTCCAACGACACTGACTTCTGACAGGATTCTTGACAGATGTTTCATATGAAGAGATGGTTAAGTCACGATAATCAATAGGAGAAATCAATATGATCGTTTCTGACATCGAAGCTAACGCCCTCTTAGAGAGCGTCACTAAGTTCCACTGCGGGGTTATCTACGACTACTCCACCGCTGAGTACGTAAGCTACCGTCCGAGTGACTTCGGTGCGTATCTGGATGCGCTGGAAGCCGAGGTctgtcgcttatac                                                                                  >  1:652464/1‑288`  
                                                                                                                                                                                                                                 `tataagagacagGGGGAAGAAGAGTCACACTCAGAGTCAACAGACTGGTCCAACGACACTGACTTCTGACAGGATTCTTGACAGTTGTTTCATATGAAGAGATTGTTAAGTCACGATAATCAATAGGAGAAATCAATATGATCATTTCTGACATCGAAGCTAACGCCCTCTTAGAGAGCGTCACTAAGTTCCACTGCGGGGTTATCTACGACTACTCCACCGCTGAGTACGTAAGCTACCGTCCGAGTGACTTCGGTGCGTATCTGGATGCGCTGGAAGCCGAGGTTGCACGAGGCGGTCT                                                                                <  1:671814/289‑1`  
                                                                                                                                                                                                                                    `ggggctcggagatgtgtataagagacagCAGAGTCAACAGACTGGTCCAACGACACTGACTTCTGACAGGATTCTTGACAGTTGTTTCATATGAAGAGATTGTTAAGTCACGATAATCAATAGGAGAAATCAATATGATCGTTTCTGACATCGAAGCTAACGCCCTCTTAGAGAGCGTAACTAAGTTCAACTGCGGGGTTATCTACGACTACTCCACCGCTGAGTACGTAAGCTACCGTCCGAGTGACTTCGGTGCGTATCTGGATGCGCTGGAAGCCGAGGTTGCACGAGGCGGTCTTAT                                                                             <  1:726032/273‑1`  
                                                                                                                                                                                                                                            `tgtgtataagagacagCACTCAGAGTCAACAGACTGGTCCAACGACACTGACTTCTGACAGGATTCTTGACAGTTGTTTCATATGAAGAGATTGTTAAGTCACGATAATCAATAGGAGAAATCAATATGATCGTTTCTGACATCGAAGCTAACGCCCTCTTAGAGAGCGTCACTAAGTTCCACTGCGGGGTTATCTACGACTACTCCACCGCTGAGTACGTAAGCTACCGTCCGAGTGACTTCGGTGCGTATCTGGATGCGCTGGAAGCCGAGGTTGCACGAGGCGGTCTTATTGTGTTCC                                                                     <  1:543877/285‑1`  
                                                                                                                                                                                                                                            `AGGGGAAGAAGAGTCACACTCAGAGTCAACAGACTGGTCCAACGACACTGACTTCTGACAGGATTCTTGACAGTTGTTTCATATGAAGAGATTGTTAAGTCACGATAATCAATATGAGAAATCAATATGATCGTTTCTGACATCGAAGCTAACGCCCTCTTAGAGAGCGTCACTAAGTTCCACTGCGGGGTTATCTACGACTACTCCACCGCTGAGTACGTAAGCTACCGTCCGAGTGACTTCGGTGCGTATCTGGATGCGCTGGAAGCCGAGGTTGCACGAGGCGGTCTTATTGTGTTCC                                                                     <  1:816728/301‑1`  
                                                                                                                                                                                                                                              `GGGAAGAAGAGTCACACTCAGAGTCAACAGACTGGTCAAACGACACTGACTTCTGACAGGATTCTTGACAGTTGTTTCATATGAAGAGATTGTTAAGTCACGATAATCAATAGGAGAAATCAATATGATAGTTTCTGACATCGAAGCTAACGCCCTCTTAGAGAGCGTCACTAAGTTCCACTGCGGGGTTATCTACGACTACTCCACCGCTGAGTACGTAAGCTACCGTCCGAGTGACTTCGGTGCGTATCTGGATGCGCTGGAAGCCGAGGTTGCACGAGGCGGTCTTATTGTGTTCCAC                                                                   <  1:732624/301‑1`  
                                                                                                                                                                                                                                              `GGGAAGAAGAGTAACACTCAGAGTCAACAGACTGGTCCAACGACACTGACTTCTGACAGGATTCTTGACAGTTGTTTCATATGAAGAGATTGTTAAGTCACGATAATCAATAGGAGAAATCAATATGATCGTTTCTGACATCGAAGCTAACGCCCTCTTAGAGAGCGTCACTAAGTTCCACTGCGGGGTTATCTACGACTACTCCACCGCTGAGTACGTAAGCTACCGTCCGAGTGACTTCGGTGCGTATCTGGATGCGCTGGAAGCCGAGGTTGCACGctgtctcttatacacatctccg                                                                   >  1:463266/1‑279`  
                                                                                                                                                                                                                                                        `GTCACACTCAGAGTCAACAGACTGGTCCAACGACACTGACTTC‑‑‑‑‑‑‑‑‑‑‑‑TGACAGTTGTTTCATATGAAGAGATTGTTAAGTCACGATAATCAATAGGAGAAATCAATATGATCGTTTCTGACATCGAAGCTAACGCCCTCTTAGAGAGCGTCACTAAGTTCCACTGCTGGGTTATCTACGACTACTCCACCGCTGAGTACGTAAGCTACCGTCCTAGTGACTTCGGTGCGTATCTGGATGCGCTGGAAGCCGAGGTTGCACGACGCGGTCTTATTGTGTTCCACAACGGTCACAAGTATGACGTTC                                             >  1:8715/1‑301`  
                                                                                                                                                                                                                                                                 `AGAGTCAACAGACTGGTCCAACGACACTGACTTCTGACAGGTTTCTTGACAGTTGTTTCATATGAAGAGATTGTTAAGTCACGATAATCAATAGGAGAAATCAATATGATCGTTTCTGACATCGAAGCTAACGCCCTCTTAGAGAGCGTCACTAAGTTCAACTGCGGGGTTGTCTACGACTACTCCACCGCTGAGTACGTAAGCTACCGTCCGAGTGACTTCGGTGCGTATCTGGATGCGCTGGAAGCCGAGGTTGCACGAGGAGGTCTTATTGTGTTCCACAACGGTCACAAGTATGACa                                                <  1:596810/301‑2`  
                                                                                                                                                                                                                                                                    `GTCAACAGACTGGTCCAACGACACTGACTTCTGACAGGATTCTTGACAGTTGTTTCATATGAAGAGATTGTTAAGTCACGATAATCAATAGGAGAAATCAATATGATCGTTTCTGACATCGAAGCTAACGCCCTCTTAGAGAGCGTCACTAAGTTCCACTGCGGGGTTATCTACGACTACTCCACCGCTGAGTACGTAAGCTACCGTCCGAGTGACTTCGGTGCGTATCTGGATGCGCTGGAAGCCGAGGTTTCACGAGGCGGTCTTATTGctgtctcttctacacatctccgaccccacc                                             >  1:807533/1‑271`  
                                                                                                                                                                                                                                                                     `TCAACAGACTGGTCCAACGACACTGACTTCTGACAGGATTCTTGACAGTTGTTTCATATGAAGAGATTGTTAAGTCACGATAATCAATAGGAGAAATCAATATGATCGTTTCTGACATCGAAGCTAACGCCCTCTTAGAGGGAGTCACTAAGTTCCACTGCGGGGTTATCTACGACTACTCCACCGCTGAGTACGTAAGCTACCGTCCGAGTGACTTCGGTGCGTATCTGGATGCGCTGGAAGCCGAGGTTGCACGAGGCGGTCTTATTGTGTTCCACAACGGTCACAAGTATGACGTTCC                                            >  1:642604/1‑301`  
                                                                                                                                                                                                                                                                          `AGACTGGTCCAACGACACTGACTTCTGACAGGATTCTTGACAGTTGTTTCATATGAAGAGATTGTTAAGTCACGATAATCAATAGGAGAAATCAATATGATCGTTTCTGACATCGAAGCTAACGCCCTCTTAGAGAGCGTCACTAAGTTCCACTGCGGGGTTATCTACGACTACTCCACCGCTGAGTACGTAAGCTACCGTCCGAGTGACTTCGGTGCGTATCTGGATGCGCTGGAAGCCGAGGTTGCACGAGGCGGTCTTATTGTGTTCCACAACGGTCACAAGTATGACGTTCCTGCAT                                       <  1:522784/301‑1`  
                                                                                                                                                                                                                                                                          `AGACTGGTCCAACGACACTGACTTCTGACAGGATTCTTGACAGTTGTTTCATATGAAGAGATTGTTAAGTCACGATAATCAATAGGAGAAATCAATATGATCGTTTCTGACATCGAAGCTAACGCCCTCGTAGAGAGCGTCACTAAGTTCCACTTCGGGGTTATCTACGACTACTCCACCGCTGAGTACGTAAGCTACCGTCCGAGTGACTTCGGTGCGTATCTGGATGCGCTGGAAGCCGAGGTTGCACGAGGCGGTCTTATTGTGTTCCACAACGGTCACAAGTATGACGTTCCTGCAT                                       <  1:608884/301‑1`  
                                                                                                                                                                                                                                                                                `GTCCAACGACACTGACTTCTGACAGGATTCTTGACAGTTGTTTCATATGAAGAGATTGTTAAGTCACGATAATCAATAGGAGAAATCAATATGATCGTTTCTGACATCGAAGCTAACGCCCTCTTAGAGAGCGTCACTAAGTTCCACTGCGGGGTTATCTACGACTACTCCACCGCTGAGTACGTAAGCTACCGTCCGAGTGACTTCGGTGCGTATCTGGATGAGCTGGAAGCCGAGGTTGCACGAGGCGGTCTTATTGTGTTCCACAACGGTCACAAGctgtctcttatacacatctccg                                 >  1:398830/1‑279`  
                                                                                                                                                                                                                                                                                    `AACGACACTGACTTCTGACAGGTTGCTTGACAGTTGTTTCATATGAAGAGATGGTTAAGTCACGATAATCAATAGGAGAAATCAATAAGATCGTTTCTGACATCGAAGCTAACGCCCTCTTAGAGAGCGTCACTAGGTTCCACTGCGGGGTTATCTACGACTACTCCACCGCTGAGTACGTAAGCTACCGTCCGAGTGACTTCGGTGCGTATCTGGATGCGCTGGAAGCCGATGTTGCACGAGGCGGTCTTATTGTGTTCCACAACGGTCACAAGTATGACGTTCCTGCATTGACCAAACT                             <  1:165709/301‑1`  
                                                                                                                                                                                                                                                                                        `ACACTGACTTCTGACAGTATTCTTGACAGTTGGTTCATATGAAGAGATTGTTAAGTCACGATAATCAATATGAGAAATCAATATGATCGTTTCTGACATCGAAGCTAACGCCCTCTTAGAGAGCGTCACTAAGTTCCACTGCGGGGTTATCTACGACTGCTCCACCGCTGAGTACGTAAGCTACCGTCCGAGTGACTTCGGTGCGTATCTGGATGCGCTGGAAGCCGAGGTTGCACGAGGCGGTCTTATTGTGTTCCACAACGGTCACAAGTATGACGTTCCTGCATTGACCAAACTGGCA                         <  1:104771/301‑1`  
                                                                                                                                                                                                                                                                                            `TGACTTCTGACAGGTTTCTTGACAGTTGTTTCATATGAAGAGATTGTTAAGTCACGATAATCAATAGGAGAAATCAATATGATCGTTTCTGACATCGAAGCTAACGCCCTCTTAGAGAGCGTCACTAAGTTCCACTGCGGGGTTATCTACGACTACTACACCGCTGAGTACGTAAGCTACCGTCCGAGTGACTTCGGTGCGTATCTGGATGCGCTGGAAGCCGAGGTTGCACGAGGCGGTCTTATTGTGTTCCACAACGGTCACAAGTATGACGTTCCTGCATTGACCAAACTGGCAAAGT                     <  1:350715/301‑1`  
                                                                                                                                                                                                                                                                                                       `AGGATTCTTGACAGTTGTTTCATATGAAGAGATTGTTAAGTCACGATAATCAATAGGAGAAATCAATATGATCGTTTCTGACATCGAAGCTAACGCCCTCTTAGAGAGCGTCACTAAGTTCCACTGCGGGGTTATCTACGACTACTCCACCGCTGAGTACGTAAGCTACCGTCCGAGTGACTTCGGTGCGTATCTGGATGCGCTGGAAGCCGAGGTTGCACGAGGCGGTCTTATTGTGTTCCACAACGGTCACAAGTATGACGTTCCTACATTGACCAAACTGGCAAAGTTGCAATTGAAC          <  1:17928/301‑1`  
                                                                                                                                                                                                                                                                                                             `CTTGACAGTTGTTTCATATGAAGAGATTGTTAAGTCACGATAATCAATAGGAGAAATCAATATGATCGTTTCTGACATCGAAGCTAACGCCCTCTTAGAGAGCGTCACTAAGTTCCACTGCGGGGTTATCTACGACTACTCCACCGATGAGTACGTAAGCTACCGTCCGAGTGACTTCGGTGCGTATCTGGATGCGCTGGAAGCCGAGGTTGCACGAGGCGGTCTTATTGTGTTCCACAACGGTCACAAGTATGACGTTCCTGCATTGACCAAACTGGCAAAGTTGCAATTGAACCGAGAG    >  1:465943/1‑301`  

                                                                                                                                                                                                                                                                                                                                                                                                                                                                                                                                                                                                                              
`GACAAAGGTAAAGCACATGAGGAAGGTCGCCCCGTTTCTATTACTGACCTACGTGGTTCTGGCGCACTACGCCAACTATCTGATACTATTATTGCCCTTGAGCGTAATCAGCAAGGCGATATGCCTAACCTTGTCCTCGTTCGTATTCTCAAGTGCCGCTTTACTGGTGATACTGGTATCGCTGGCTACATGGAATACAACAAGGAAACCGGATGGCTTGAACCATCAAGTTACTCAGGGGAAGAAGAGTCACACTCAGAGTCAACAGACTGGTCCAACGACACTGACTTCTGACAGGATTCTTGA‑‑‑‑‑‑‑‑‑‑‑‑‑‑‑‑‑‑‑‑‑‑‑‑‑‑‑‑‑‑‑‑‑‑‑‑‑‑‑‑‑‑‑‑‑‑‑‑‑‑‑‑‑‑‑‑‑‑‑‑‑‑‑‑‑‑‑‑‑‑‑‑‑‑‑‑‑‑‑‑‑‑‑‑‑‑‑‑‑‑‑‑‑‑‑‑‑‑‑‑‑‑‑‑‑‑‑‑‑‑‑‑‑‑‑‑‑‑‑‑‑‑‑‑‑‑‑‑‑‑‑‑‑‑‑‑‑‑‑‑‑‑‑‑‑‑‑‑‑‑‑‑‑‑‑‑‑‑‑‑‑‑‑‑‑‑‑‑‑‑‑‑‑‑‑‑‑‑‑‑‑‑‑‑‑‑‑‑‑‑‑‑‑‑‑‑‑‑‑‑‑‑‑‑‑‑‑‑‑‑‑‑‑‑‑‑‑‑‑‑‑‑‑‑‑‑‑‑‑‑‑‑‑‑‑‑‑‑‑‑‑‑‑‑‑‑‑‑‑‑‑‑‑‑‑‑‑‑‑‑‑‑‑‑‑‑‑‑‑‑‑‑‑‑‑‑‑‑‑‑‑‑‑‑‑‑‑‑‑‑‑‑‑‑‑‑‑‑  >  NC_001604.1/12972‑13277`  
`‑‑‑‑‑‑‑‑‑‑‑‑‑‑‑‑‑‑‑‑‑‑‑‑‑‑‑‑‑‑‑‑‑‑‑‑‑‑‑‑‑‑‑‑‑‑‑‑‑‑‑‑‑‑‑‑‑‑‑‑‑‑‑‑‑‑‑‑‑‑‑‑‑‑‑‑‑‑‑‑‑‑‑‑‑‑‑‑‑‑‑‑‑‑‑‑‑‑‑‑‑‑‑‑‑‑‑‑‑‑‑‑‑‑‑‑‑‑‑‑‑‑‑‑‑‑‑‑‑‑‑‑‑‑‑‑‑‑‑‑‑‑‑‑‑‑‑‑‑‑‑‑‑‑‑‑‑‑‑‑‑‑‑‑‑‑‑‑‑‑‑‑‑‑‑‑‑‑‑‑‑‑‑‑‑‑‑‑‑‑‑‑‑‑‑‑‑‑‑‑‑‑‑‑‑‑‑‑‑‑‑‑‑‑‑‑‑‑‑‑‑‑‑‑‑‑‑‑‑‑‑‑‑‑‑‑‑‑‑‑‑‑‑‑‑‑‑‑‑‑‑‑‑‑‑‑‑‑‑‑‑‑‑‑‑‑‑‑‑‑‑‑‑‑‑‑‑‑‑‑‑‑‑‑‑‑‑‑‑‑‑‑‑‑‑‑‑‑‑ttcttgaCAGTTGTTTCATATGAAGAGATTGTTAAGTCACGATAATCAATAGGAGAAATCAATATGATCGTTTCTGACATCGAAGCTAACGCCCTCTTAGAGAGCGTCACTAAGTTCCACTGCGGGGTTATCTACGACTACTCCACCGCTGAGTACGTAAGCTACCGTCCGAGTGACTTCGGTGCGTATCTGGATGCGCTGGAAGCCGAGGTTGCACGAGGCGGTCTTATTGTGTTCCACAACGGTCACAAGTATGACGTTCCTGCATTGACCAAACTGGCAAAGTTGCAATTGAACCGAGAGTT  >  NC_001604.1/14297‑14594`

  

Alignment Legend

Aligned base mismatch/match (shaded by quality score): `ATCG/ATCG` `< 3 ≤` `ATCG/ATCG` `< 16 ≤` `ATCG/ATCG` `< 28 ≤` `ATCG/ATCG` `< 37 ≤` `ATCG/ATCG` `< 38 ≤` `ATCG/ATCG`

Unaligned base: `atcg`    Masked matching base: `atcg`    Alignment gap: `‑`    Deleted base: `‑`

Reads not counted as support for junction

read\_name Not counted due to insufficient overlap past the breakpoint.

read\_name Not counted due to not crossing MOB target site duplication.
